# Thr105Ile (rs11558538) polymorphism in the histamine *N*-methyltransferase (*HNMT*) gene and risk for Parkinson disease: A PRISMA-compliant systematic review and meta-analysis

**DOI:** 10.1097/MD.0000000000004147

**Published:** 2016-07-08

**Authors:** Félix Javier Jiménez-Jiménez, Hortensia Alonso-Navarro, Elena García-Martín, José A.G. Agúndez

**Affiliations:** a Section of Neurology, Hospital Universitario del Sureste, Arganda del Rey; b Department of Medicine-Neurology, Hospital “Príncipe de Asturias,” Universidad de Alcalá, Alcalá de Henares, Madrid; c Department of Pharmacology, University of Extremadura, Cáceres, Spain.

**Keywords:** genetics, HNMT polymorphisms, meta-analysis, Parkinson disease

## Abstract

**Background/aims:**

Several neuropathological, biochemical, and pharmacological data suggested a possible role of histamine in the etiopathogenesis of Parkinson disease (PD). The single nucleotide polymorphism (SNP) rs11558538 in the histamine *N*-methyltransferase (*HNMT*) gene has been associated with the risk of developing PD by several studies but not by some others. We carried out a systematic review that included all the studies published on PD risk related to the rs11558538 SNP, and we conducted a meta-analysis following Preferred Reporting Items for Systematic Reviews and Meta-Analyses guidelines.

**Methods:**

We used several databases to perform the systematic review, the software *Meta-DiSc 1.1.1* to perform the meta-analysis of the eligible studies, and the Q-statistic to test heterogeneity between studies.

**Results:**

The meta-analysis included 4 eligible case–control association studies for the *HNMT* rs11558538 SNP and the risk for PD (2108 patients, 2158 controls). The frequency of the minor allele positivity showed a statistically significant association with a decreased risk for PD, both in the total series and in Caucasians. Although homozygosity for the minor allele did not reach statistical significance, the test for trend indicates the occurrence of a gene–dose effect. Global diagnostic odds ratios (95% confidence intervals) for rs11558538T were 0.61 (0.46–0.81) for the total group, and 0.63 (0.45–0.88) for Caucasian patients.

**Conclusion:**

The present meta-analysis confirms published evidence suggesting that the *HNMT* rs11558538 minor allele is related to a reduced risk of developing PD.

## 1 Introduction

Histamine is stored and released in the brain, mainly in mast cells and histaminergic neurons of the tuberomammilary nucleus of the posterior basal hypothalamus. Histaminergic fibers project from this nucleus to many regions of the brain, including the striatum, thalamus, cerebral cortex, hippocampus, and amygdale.^[1,2]^ Histamine acts through 4 metabotropic histamine receptors (HRs), designated as HRH1, HRH2, HRH3, and HRH, which are all G-protein-coupled. HRH3 is implicated in neurotransmitter release in the central nervous system. Histidine decarboxylase is the enzyme responsible for the synthesis of histamine (from its precursor histidine), whereas histamine *N*-methyltransferase (HNMT) and diamine oxidase (or ABP1) are the responsible enzymes in inactivating histamine, respectively, in the brain and in the peripheral tissues (revised in Ref.^[3]^).

Together with the demonstration that histamine infusion could induce neuronal death and inflammatory phenomena in the *substantia nigra* of rats,^[4]^ recent neuropathological, biochemical, and pharmacological data arisen from studies in patients with Parkinson disease (PD), and in experimental models of parkinsonism, which are summarized in Table 1, suggest an implication of histamine in the pathogenesis of PD.^[5–24]^

**Table 1:**
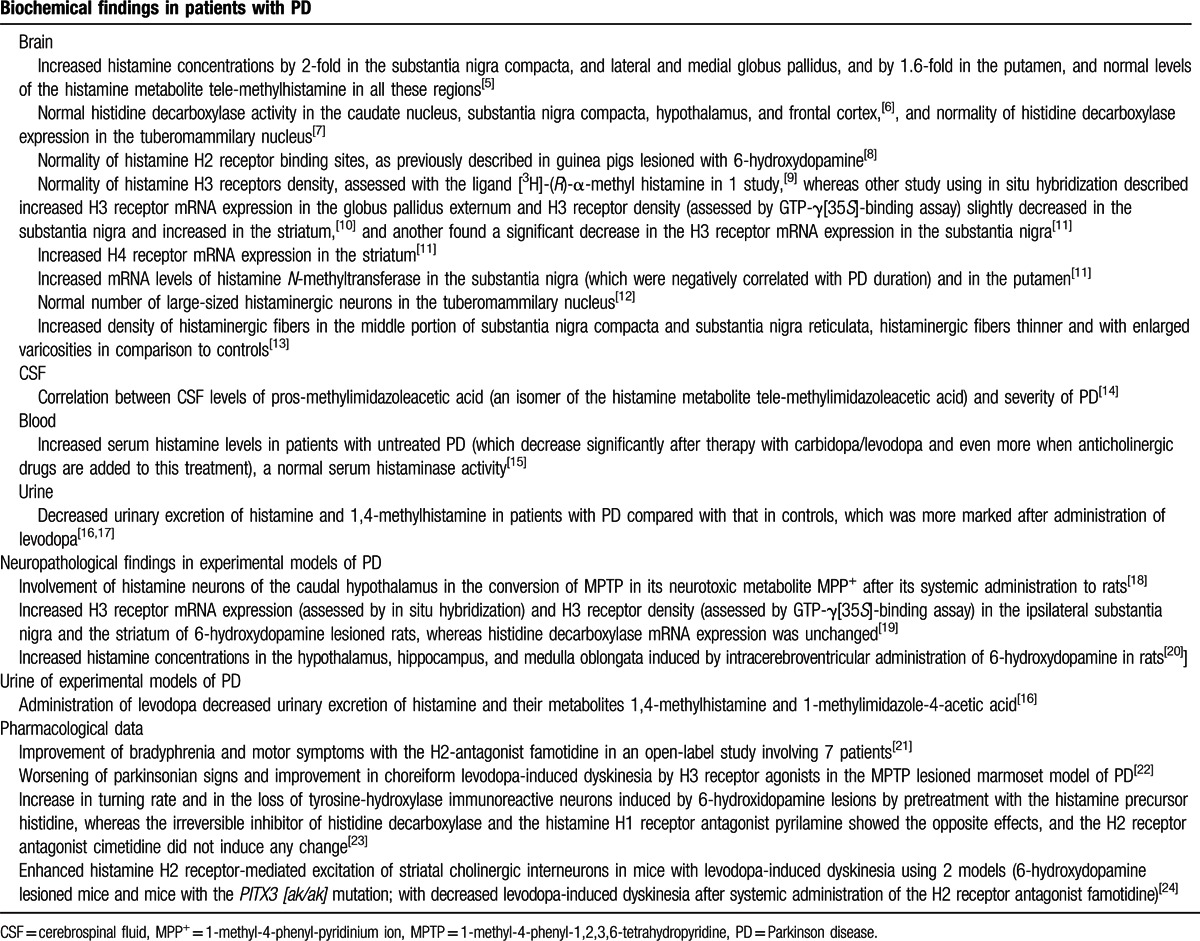
Results of studies on brain, CSF, blood, and urine of histamine-related substances in patients with PD, neuropathological data in models of parkinsonism, and pharmacological data in patients with PD and experimental models of parkinsonism related to histamine.

The single nucleotide polymorphism (SNP) rs11558538, located in the exon 4 C314T of the *HNMT* gene (chromosome 2q22.1, MIM 605283, gene identity 3176), which causes the amino acid substitution Thr105Ile (related to decreased enzyme activity), has been the matter of several case–control association studies trying to establish its association with the risk of developing PD. However, the results of studies addressing this association have been controversial. For this reason, we performed a systematic review and a meta-analysis of eligible studies, including an estimation of the genetic association of each study and of the pooled data, and investigated the heterogeneity between studies.

## 2 Materials and methods

### 2.1 Search strategy

**Figure 1:**
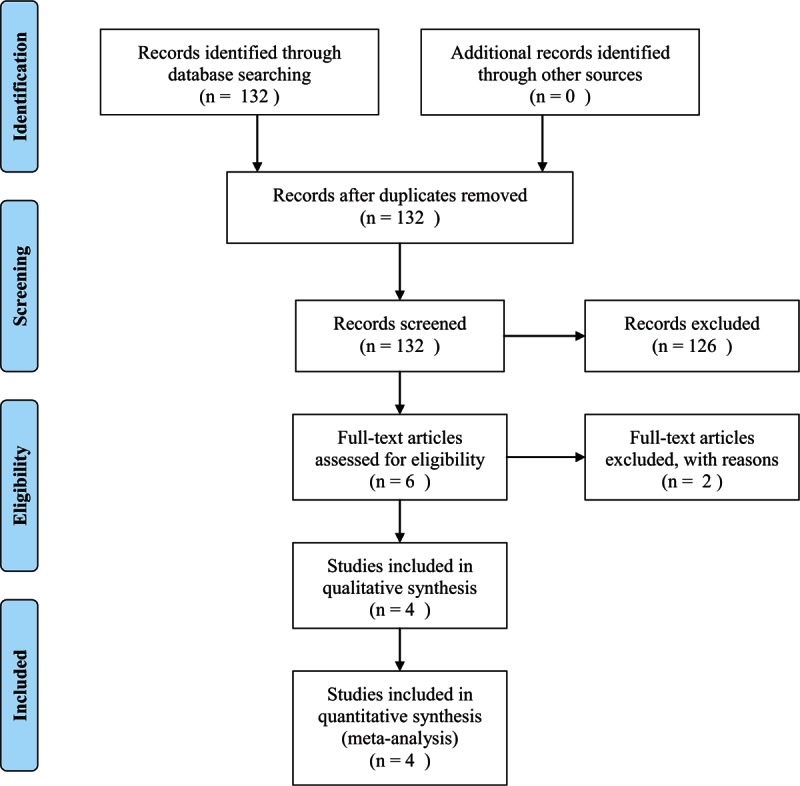
Flowchart of the study selection.

Figure 1 shows the literature search and selection of eligible studies. We crossed the terms “histamine”, “HNMT,” and “HNMT gene” with “Parkinson's disease,” using several databases, to identify the eligible studies for the systematic review and meta-analysis. The search, which included all publications in any language, during the period from 1966 to November 24, 2015, retrieved the following results: PubMed (130 reports), MEDLINE Plus (0 report), EMBASE (38 reports), Science Citation Index Expanded (0 report), National Institute for Health and Care Excellence (0 report), and Cochrane Central Register of Controlled Trials in the Cochrane Library (0 report). We also consulted the PD Gene Data Base (link: http://archive.pdgene.org/). The search using these databases showed 6 case–control association studies on rs11558538 SNPs in the *HNMT* gene and the risk for PD.^[25–30]^ However, we excluded from the review and meta-analyses 2 studies included in the PD Gene Data Base because the genotype and allele frequencies of the analyzed SNP were not available in the respective reports.^[26,27]^ Therefore, only 4 case–control studies were included in the final meta-analysis.^[25,28–30]^

### 2.2 Data extraction and analysis

We extracted the following information: journal, publication year, first author, number of cases and controls for each *HNMT* rs11558538 genotype, genotyping method, and demographics, and we calculated allele frequencies, from each study. We also analyzed the statistical significance of the association of *HNMT* rs11558538 alleles and the risk of developing PD for each study to avoid statistical inconsistencies, and indicated all associations as diagnostic odds ratios (ORs) with their corresponding 95% confidence intervals (CIs). Fixed effects of the pooled OR, as well as random pooled OR effects, were estimated, based on individual ORs.

### 2.3 Statistical analysis

The meta-analyses of the eligible case–control studies regarding association between rs11558538 SNP and PD risk were carried out by using the software *Meta-DiSc 1.1.1* (http://www.hrc.es/investigacion/metadisc.html; Unit of Clinical Statistics, Hospital Ramón y Cajal, Madrid, Spain).^[31]^ The Mantel–Haenszel^[32]^ and the DerSimonian–Laird methods^[33]^ were used, respectively, to calculate the global diagnostic OR when no heterogeneity was observed and when statistically significant heterogeneity existed.

The Q-statistic was used to test heterogeneity between studies.^[34]^ Heterogeneity was considered significant when *P* < 0.10, and was quantified by using the I^2^ metric (I^2^ = [Q] degrees of freedom [d.f.])/Q), which is independent of the number of studies included in the meta-analysis.^[35]^ I^2^ takes values between 0% and 100%, with higher values denoting greater degree of heterogeneity. We also calculated the statistical power for the pooled sample of the eligible studies.

Because this study is a systematic review and meta-analysis, ethical approval was not needed. This work was elaborated according to the Preferred Reporting Items for Systematic Reviews and Meta-Analyses guidelines.^[36]^

## 3 Results

The meta-analysis included a total of 4 eligible studies analyzing the association between the *HNMT* rs1155858 SNP and the risk for PD (2108 patients with PD, 2158 controls). The genotype distribution and the minor allele frequencies from patients with PD and control groups in the eligible studies are summarized in Tables 2 and 3, respectively.

**Table 2:**
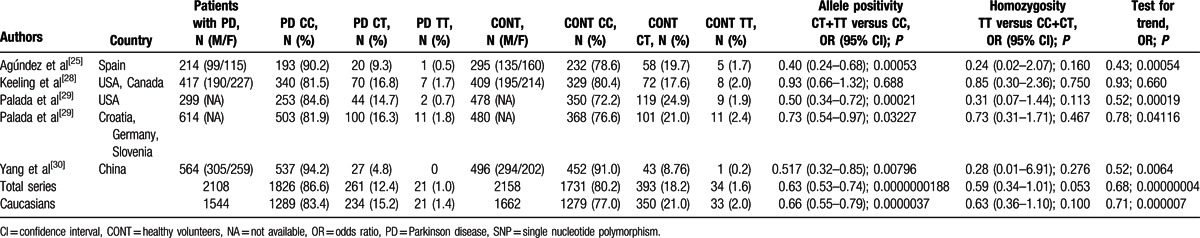
Frequency of the genotypes (CC, CT, and TT) of the SNP rs11558538 in the *HNMT* gene in the total series of patients with PD and CONT in different reports with their ORs and 95% CIs.

**Table 3:**
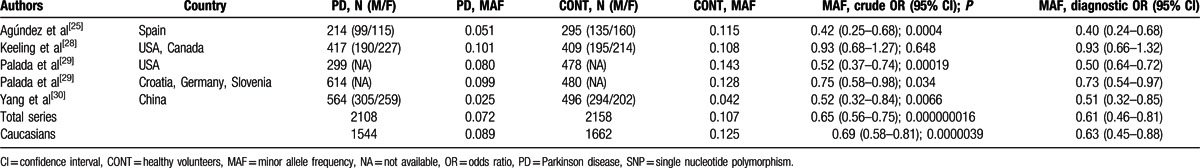
Frequency of the allelic variants of the SNP rs11558538 in the *HNMT* gene in the total series of patients with PD and CONT in different reports with their diagnostic ORs and 95% CIs.

**Figure 2:**
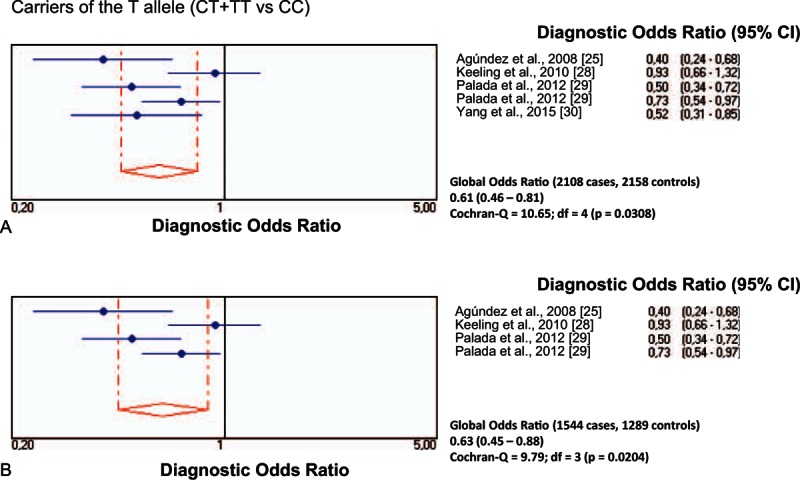
Diagnostic odds ratios and 95% CIs for each study and for pooled samples for carriers of the rs11558538T allele in patients with PD and controls in total series (A) and in Caucasian patients (B). CI = confidence interval, PD = Parkinson disease.

**Figure 3:**
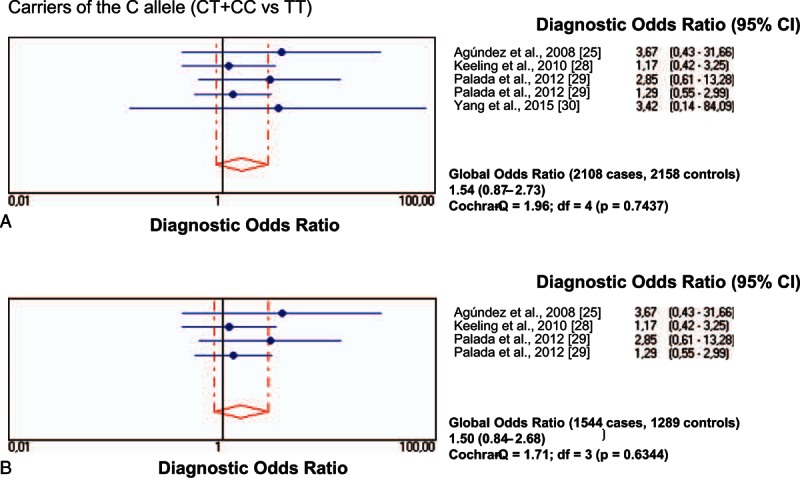
Diagnostic odds ratios and 95% CIs for each study and for pooled samples for carriers of the rs11558538C allele in patients with PD and controls in total series (A) and in Caucasian patients (B). CI = confidence interval, PD = Parkinson disease.

All individual studies on the rs11558538 SNP in PD were at Hardy–Weinberg equilibrium both in the patients with PD and in the control group. The frequency of allele T positivity (CT+TT vs CC) showed a significant association between *HNMT* rs11558538 and the risk for PD, both in the total series and in the series confined to Caucasian populations (Table 2; Fig. 2A and B), whereas homozygosity (TT vs CC+CT) did not show association (Table 2; Fig. 3A and B).

**Figure 4:**
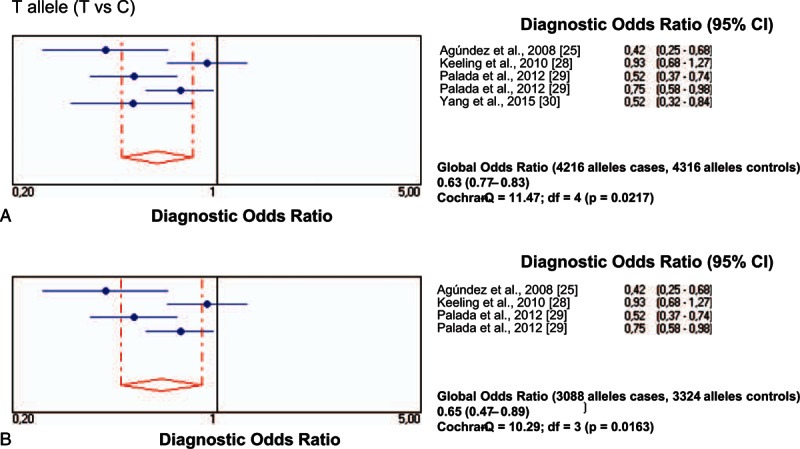
Diagnostic odds ratios and 95% CIs for each study and for pooled samples of rs11558538T allele (minor allele) in patients with PD and controls in total series (A) and in Caucasian patients (B). CI = confidence interval, PD = Parkinson disease.

**Figure 5:**
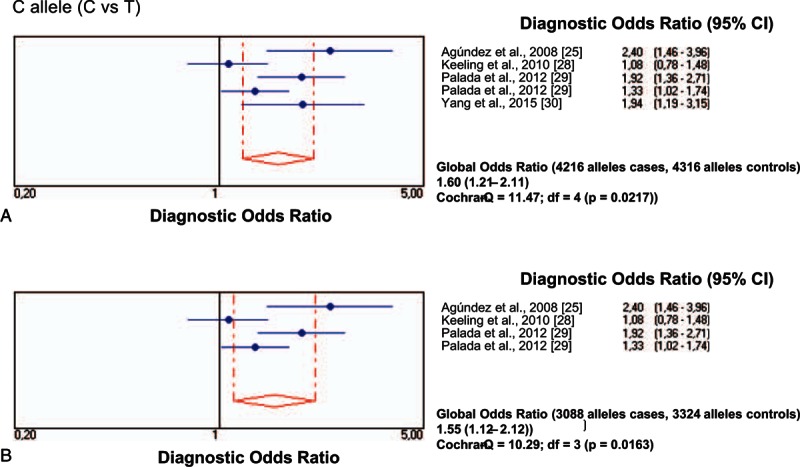
Diagnostic odds ratios and 95% CIs for each study and for pooled samples of rs11558538C allele (major allele) in patients with PD and controls in total series (A) and in Caucasian patients (B). CI = confidence interval, PD = Parkinson disease.

Figure 4A and B represents the results of the diagnostic OR and the 95% CI of all the studies and the pooled samples, which show a significant association between the minor allele of *HNMT* rs11558538 and the risk for PD, both in the total group (Fig. 4A) and in Caucasian patients (Fig. 4A). The diagnostic OR and the 95% CI of the major alleles, represented in Fig. 5A and B for the total series and Caucasians, respectively, showed a milder, although significant, association between the major allele of *HNMT* rs11558538 and the risk for PD as well. Q-statistic did show a marginally significant heterogeneity between studies, which was due to that by Keeling et al.^[28]^

The statistical power for the presence of the SNP analyzed in this study was determined from the variant allele frequencies observed in control individuals with a genetic model analyzing the frequency for carriers of the disease gene with a relative risk value = 0.65 (*P* = 0.05). For overall (Caucasian and Asian) patients the power is equal to 99.6% for 1-tailed association and 99.0% for 2-tailed association, and for Caucasian patients it was 97.9% for 1-tailed association and 95.7% for 2-tailed association.

## 4 Discussion

To date, at least 28 susceptibility loci associated with the risk for PD have been identified in genome-wide association studies (GWAS), the strongest associations related to polymorphisms being in the *MAPT*, *SNCA*, *HLA-DQB1*, *GBA*, *SYT11*, and *GAK-DGKQ* genes, but other genes such as *CCDC62/HIP1R*, *MCCC1/LAMP3*, *ACMSD*, *STK39*, *STX1B*, *RIT2*, and *BST1* have also been found to be associated with the modification of PD risk.^[37]^ Interestingly, meta-analyses of case–control association studies involving SNPs in candidate genes that were not mentioned as possible susceptibility genes in GWAS showed strong associations of many of them with the risk for PD (revised in Ref.^[37]^). Data suggesting the possible implication of histamine (summarized in Table 1) in the pathogenesis of PD make reasonable the investigation of the possible role of histamine-related genes in the risk for this disease, despite the fact that none of these have been mentioned among the possible susceptibility genes in GWAS.

Our group described an association between the major allele of the rs1155838 SNP in the *HNMT* gene and the increased risk for PD.^[25]^ Two further studies, 1 involving Caucasian patients^[29]^ and other involving Asian patients,^[30]^ showed similar results, whereas another group reported lack of association.^[28]^ Case–control association studies on other histamine-related genes showed lack of association between the *ABP1* His645Asp polymorphism,^[25]^ the nonsynonymous *HRH1* SNP designated as rs2067470 (Leu449Ser),^[38]^ and the promoter *HRH2* SNP designated as rs2067474 (G1018A)^[38]^ polymorphisms and the risk for PD.

The present systematic review and meta-analysis, which included 4 studies involving 2108 patients with PD and 2158 controls, showed a significantly lower frequency of patients carrying the minor allele of the *HNMT* rs11558538 SNP in patients with PD than in controls, both in the allele positivity analysis and in the comparison of minor allele frequencies, whereas the association of *HNMT* rs11558538TT homozygosity with PD risk did not reach statistical significance because of the low frequency of the homozygous genotype. Nevertheless, the high significance of the test for trend with the number of minor alleles (Table 2) strongly suggests the occurrence of a gene–dose effect.

The mechanism by which HNMT inactivates histamine consists in the transference of a methyl group from *S*-adenosyl-l-methionine (AdoMet) to the N_∊2_ atom of the imidazole ring, which results in the production of the histamine inactive metabolite *N*-methylhistamine and *S*-adenosyl-l-homocysteine (AdoHcy).^[39]^ Pang et al^[40]^ showed, using a theoretical 3D model of human HNMT, that the polymorphic residue Thr105Ile is located in the turn between an α helix and a β strand on the protein surface away from the active site of HNMT, and that the presence of Ile105 caused destabilization of folded HNMT, leading to the formation of a misfolded protein that is cleared by proteasomes, and therefore to a decreased enzymatic activity. The decreased activity of HNMT in patients carrying the rs11558538 minor allele should hypothetically lead to an increase in brain histamine levels, an increase in brain mRNA levels of HNMT, or both.^[41]^ The results of the present meta-analysis suggest that decreased histamine metabolism in the central nervous system should play a protective role against development of PD.

Despite the fact that our study has as the main limitation the relatively low number of studies on the association between *HNMT* rs11558538 SNP and PD risk that fulfill inclusion criteria, our data point at a protective role of the *HNMT* rs11558538T variant on the risk of developing PD (the calculated statistical power for the mean OR for carriers of the minor allele—0.65—seems to be acceptable), and give support to the hypothesis of a possible role of histamine in the pathogenesis of this disease.
